# Socially Assistive Devices in Healthcare–a Systematic Review of Empirical Evidence from an Ethical Perspective

**DOI:** 10.1007/s11948-022-00419-9

**Published:** 2023-02-02

**Authors:** Joschka Haltaufderheide, Annika Lucht, Christoph Strünck, Jochen Vollmann

**Affiliations:** 1 Institute for Medical Ethics and History of Medicine, Ruhr-University Bochum, Markstr. 258a, 44799 Bochum, Germany; 2 School of Life Sciences, University of Siegen, Siegen, Germany; 3 Institute of Gerontology, Technical University Dortmund, Dortmund, Germany

**Keywords:** Health care technology, Health services for the aged, Medical ethics, Systematic review

## Abstract

Socially assistive devices such as care robots or companions have been advocated as a promising tool in elderly care in Western healthcare systems. Ethical debates indicate various challenges. An important part of the ethical evaluation is to understand how users interact with these devices and how interaction influences users’ perceptions and their ability to express themselves. In this review, we report and critically appraise findings of non-comparative empirical studies with regard to these effects from an ethical perspective.

Electronic databases and other sources were queried using a comprehensive search strategy generating 9851 records. Studies were screened independently by two authors. Methodological quality of studies was assessed. For 22 reports on 21 datasets using a non-comparative design a narrative synthesis was performed.

Data shows positive findings in regard to attitudes and emotional reactions of users. Varying perception of a social relation and social presence are the most commonly observed traits of interaction. Users struggle with understanding technical complexities while functionality of the devices is limited. This leads to a behavioral alignment of users towards the requirements of the devices to be able to make use of them.

This evidence adds to three important ethical debates on the use of socially assistive devices in healthcare in regard to (1) reliability of existing empirical evidence to inform normative judgements, (2) ethical significance of the social presence of devices and (3) user autonomy in regard to behavioral alignment.

## Background

Socially assistive technologies (SATs) are increasingly used in healthcare. This includes, for example, various types of care robots, smart screen assistants, virtual avatars, or companion devices (Abdi et al., 2018; Leng et al., 2019; Mordoch et al., 2013; Noy et al., 2013; Yusif et al., 2016). Their use is discussed as a potential way to increase and maintain autonomy in caring situations (Bennett et al., 2017; Cowan and Turner-Smith 1999). Although no common definition exists, we understand this class of devices to entail three essential features that define a relatively new technical concept for care. First, SATs integrate into the daily environment of their users and accompany and support daily activities. In doing so, their purpose is, secondly, to provide support by addressing or handling routine, controlling or steering tasks, and by interacting with or on behalf of their users (Abdi et al., 2018; Feil-Seifer & Mataric, 2005). Thirdly and most importantly, these devices have in common that services are provided through interfaces that resemble interaction with animate beings. This can include, for example, anthropomorphic or zoomorphic design, mimicking of behavior or the display of emotional states, wishes, and desires as well as ways of communicating, for example, by use of natural language (Breazeal et al., 2016; Hegel et al., 2009). To display such kinds of outputs and states, SATs often use advanced digitized technologies to detect actions and reactions of their users such as face or gesture recognition or modeling of emotional states to react accordingly. SATs, hence, enrich technical interaction with an emotional or social dimension providing a kind of interaction that resembles more intuitive ways of human behavior (Shaw-Garlock, 2011). This allows to easily access technical functions and to provide complex supportive services on different levels.

A common field of application is in caring for elderly or frail persons. From a societal and ethical perspective, this has to be understood with reference to the larger picture of e-health and against the background of the demographic development, especially in western societies (Moerenhout et al., 2018). Given the rapid aging of these societies, it is assumed that available resources will not suffice to satisfy the needs for support and care of an increasingly aging population (Bemelmans et al., 2011; Shishehgar et al., 2018). In this regard, SATs promise to provide a more efficient way of care delivery allowing users to maintain independence and autonomy despite growing limitations while at the same time relieving care providers of more simple and repetitive tasks and free resources for high-quality care (Bemelmans et al., 2011).

Connected to this promise is a variety of ethical problems. Arguments in favor of SAT’s highlight a tailored fit between services provided and elderly peoples’ needs (Shishehgar et al., 2018). They emphasize the ethical importance of autonomy, individual freedom, and societal participation (Vandemeulebroucke et al., 2018b). Critical voices claim that SATs challenge long-standing caring practices based on arguments of efficiency, sacrificing the value of human contact over a technical rationalization of care processes (Sharkey & Sharkey, 2012; Sparrow & Sparrow, 2006). In addition, the social interface of SATs has raised concerns in regard to possible infantilization of users, their probable deception, or a loss of autonomy due to SATs deeply integrating into the everyday life and silently winning control as a technical background pacemaker (Danaher, 2020; Matthias, 2015).

Most of the existing ethical research on SATs in healthcare applies an instrumental view of technology (Vandemeulebroucke et al., 2018b). Instrumental views are defined by understanding technological devices as closed entities whose meaning and significance is defined by their intended functionality (Verbeek, 2006). Accordingly, this view investigates technology as a passive object used at humans’ will. Ethical evaluation, hence, confines itself to functional purpose, adequacy of technology as a means, and requirements of responsible use (Vandemeulebroucke et al., 2018a). In light of the recent developments in philosophy of technology, this view has to be criticized as being too narrow and treating technological artifacts merely as “black boxes”. Following this criticism, it has been suggested to view technologies as social phenomena, establishing complex relations with their users as mediators between humans and their lifeworld, thereby shaping their way of being able to perceive and express themselves within this world (Aydin et al., 2019; Verbeek, 2015). This adds an important dimension to the ethical considerations in regard to SATs. It, first, raises the question of whether and to what extent relations with SATs as instances of certain technologies change the way users perceive and are able to express themselves in their lifeworld. Secondly, it raises the question of whether and to what extent these changes are ethically acceptable (Verbeek 2006).

As the first question indicates, a prerequisite for such empirically informed judgments is extensive knowledge about the actual relational and mediating effects of SATs to be able to develop an informed perspective. However, existing empirical knowledge is scarce and vastly dispersed over different academic fields including computer science, social sciences, medicine, and nursing sciences. Against this background, we conducted a comprehensive systematic review to gather all available empirical evidence in line with the following aims: (1) To identify existing data in regard to relational effects of SATs in older adults in healthcare (2) To develop an overview of the existing methodologies to gather respective data and to critically appraise validity of existing evidence (3) To analyze existing data from an ethical perspective, to assess its fruitfulness for informing ethical arguments and to identify empirical research gaps from an ethical perspective.

In this paper, we will report on results from qualitative, descriptive quantitative, and mixed-methods studies, that is, non-comparative study designs of all sorts. Results from comparative studies such as before-after-studies, controlled trials, or randomized controlled trials will be reported separately. This decision follows from the methodologically relevant differences between comparative and non-comparative study types and the empirical statements they generate. From a health research perspective, these two types of study designs are often viewed as merely presenting a difference in quality and reliability of generated knowledge. From an ethical perspective, however, it is important that these study types also pursue different goals (to compare phenomena to another; to describe a phenomenon qualitatively, quantitatively, or both) and, hence, generate different kinds of empirical knowledge (Lau & Kuziemsky, 2016), allowing for different types of arguments. While, for example, consequentialist ethical arguments heavily rely on comparative knowledge, deontological arguments tend to reference intrinsic properties of ethically relevant entities as generated by observations in non-comparative studies.

## Methods

### Review Design

The protocol was designed and agreed upon by the authors. It was registered in the international prospective register of systematic reviews (ID CRD42020160853). It includes a systematic approach of gathering all available empirical evidence regarding effects and perceptions of human-machine interaction with SATs in healthcare. We first screened relevant databases. Articles were then retrieved and screened, based on a set of operationalized inclusion and exclusion criteria. These were drawn from our initial considerations in regard to the population of older adults, the definition of SATs, and considerations regarding the settings to include. A narrative synthesis was performed.

### Inclusion and Exclusion Criteria

In accordance with the definition outlined above, we determined to focus on (a) the context of everyday use and support by SATs of (b) older adults in (c) typical care settings. Studies had to explore experiences and perceptions of users on any kind of technical device that could be seen to match the criteria. The population criterion was operationalized by checking demographic descriptions of study populations to determine whether more than half of the population was over 18 years and could be understood as being in need of care or assistance. Studies including additional groups such as caregivers or relatives were included. In these cases, only relevant material was extracted. Typical care settings should include studies in rehabilitative settings, home care settings, retirement villages, part-time care, and nursing homes. We also included experimental laboratory settings (living labs), which are commonly used in computer science to conduct studies with prototypes and devices that cannot be integrated into other environments for technical reasons. However, in regard to these types of studies, we determined that the set-up had to satisfy a typical use case comparable to the settings noted above. Publication date was set from 1970 to present.

Theoretical articles addressing technical frameworks, or considering ethical or medical issues with no reference to empirical data were excluded. We also excluded studies with devices that were teleoperated (e.g. wizard-of-oz-studies) by actual humans or were only used to connect with other persons, for example by videoconference-calls. From a methodological point of view, we excluded single case reports and single-user experiments as well as proof-of-concept studies and studies that solely evaluated the technical functions of devices as long as these studies did not include findings in regard to the users’ experiences and perceptions.

### Search

Database searches were carried out in February 2020 with an update in May 2021. Databases included were Medline via PubMed, ProQuest, ScienceDirect CINAHL, Embase, EUROETHICS, NIHR-HTA, and Cochrane Library. In addition, we searched for grey literature, citations of full-text inclusions, scanned conference proceedings, and consulted with experts from the field. Detailed information on the sources can be found in Box 1.

**Box 1:**
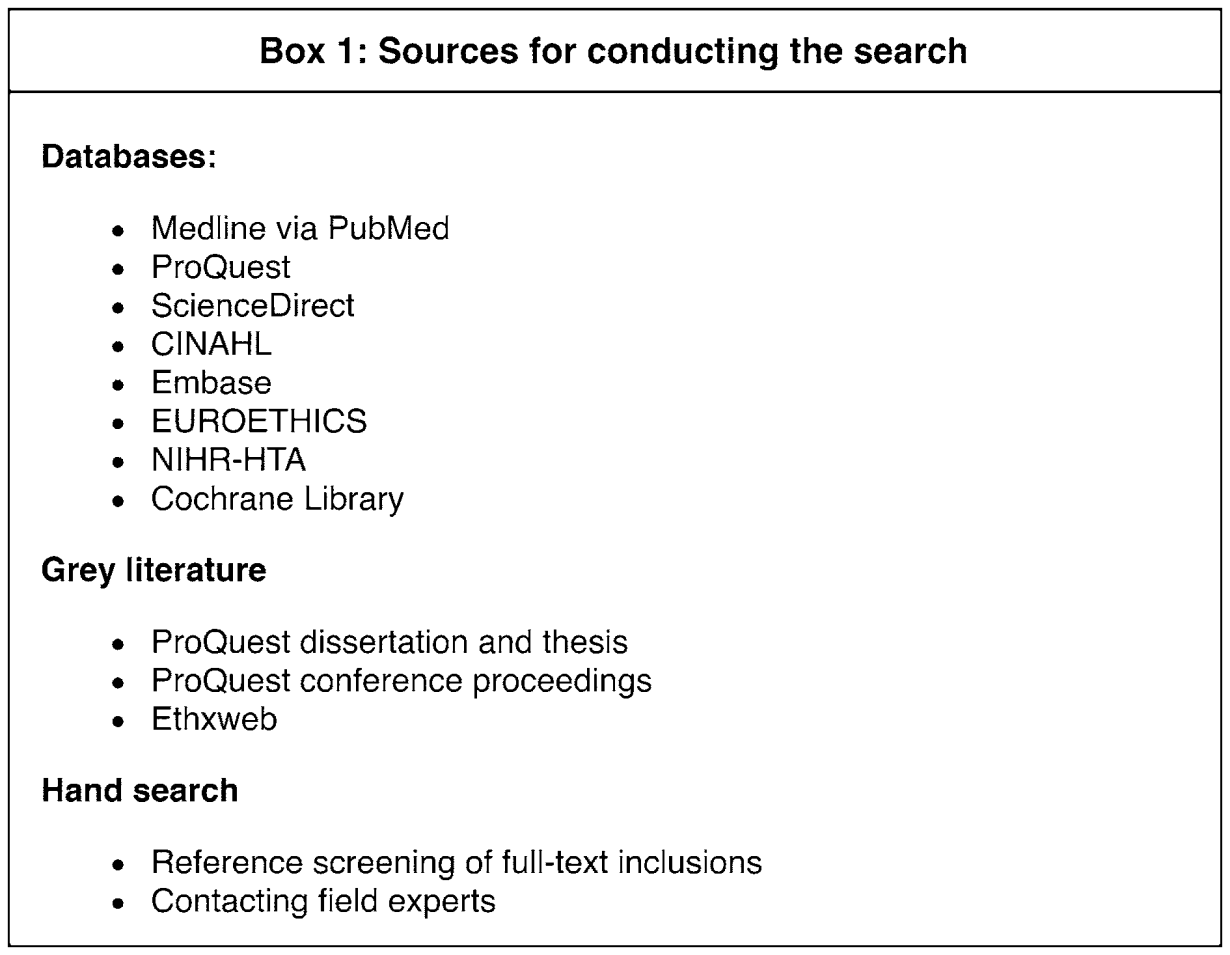
Sources for conducting the search

### Selection and Data Extraction

Three of the authors (JH, CS, AL) with the help of two assistants independently screened titles and abstracts. The assistants were supervised by the first author and their recommendations for decisions were reviewed separately. Full texts were screened by two authors (JH, AL) independently. Data was extracted independently by two authors (JH, AL) using a modified data collection form based on the template of Cochrane Foundation. Subsequently, extractions were synthesized. A detailed overview of the extracted data is given in Online Resource 1. In case of disagreement during title and abstract screening, the decision was delayed until full-text was accessed. In case of disagreement during full-text screening, a third author was consulted. Reasons for exclusion were documented. In case of missing information or uncertainty as well as in case of indications for further reports, the main authors were contacted.

### Assessment of Methodological Quality

Contrary to the original plan outlined in the protocol, we present the review of methodological quality using the MMAT tool for mixed-methods reviews (Hong et al., 2018). This follows the decision to report qualitative, quantitative, and mixed-methods studies together and is intended to provide a better overview of the quality of the studies. Appraisal of methodological quality was conducted independently by JH and AL. Disagreements were dissolved during discussion.

### Synthesis

A narrative synthesis in accordance with Pope et al. (2007) was performed as this enables the diverse studies and designs to be incorporated and interconnected. Narrative synthesis allows for specific aggregation of pre-identified core themes across diverse studies. The authors (JH, AL) independently compiled findings from extracted data by inductive development of themes. These themes were, then, synthesized in a joint coding using MAXQDA. The aim was to identify overarching “core themes” and general patterns occurring in different studies and are, thus, suitable for providing general statements, even across the diverse study base.

## Results

We retrieved 9851 records from electronic databases. After removal of duplicates, 9082 records remained. 8793 records were excluded based on title and abstract screening. After full-text screening, in total 55 electronic records remained. Additional sources revealed 10 more records. 5 of them were included. In total 60 records were included. 21 studies in 22 reports were of non-comparative design on which we will focus in this paper. The detailed flow of studies through the screening process can be seen in Fig. 11. Over all studies (comparative and non-comparative) Cohen’s kappa was 0.85 which indicates almost perfect agreement between raters in the screening process (Landis & Koch, 1977).

**Fig. 1:**
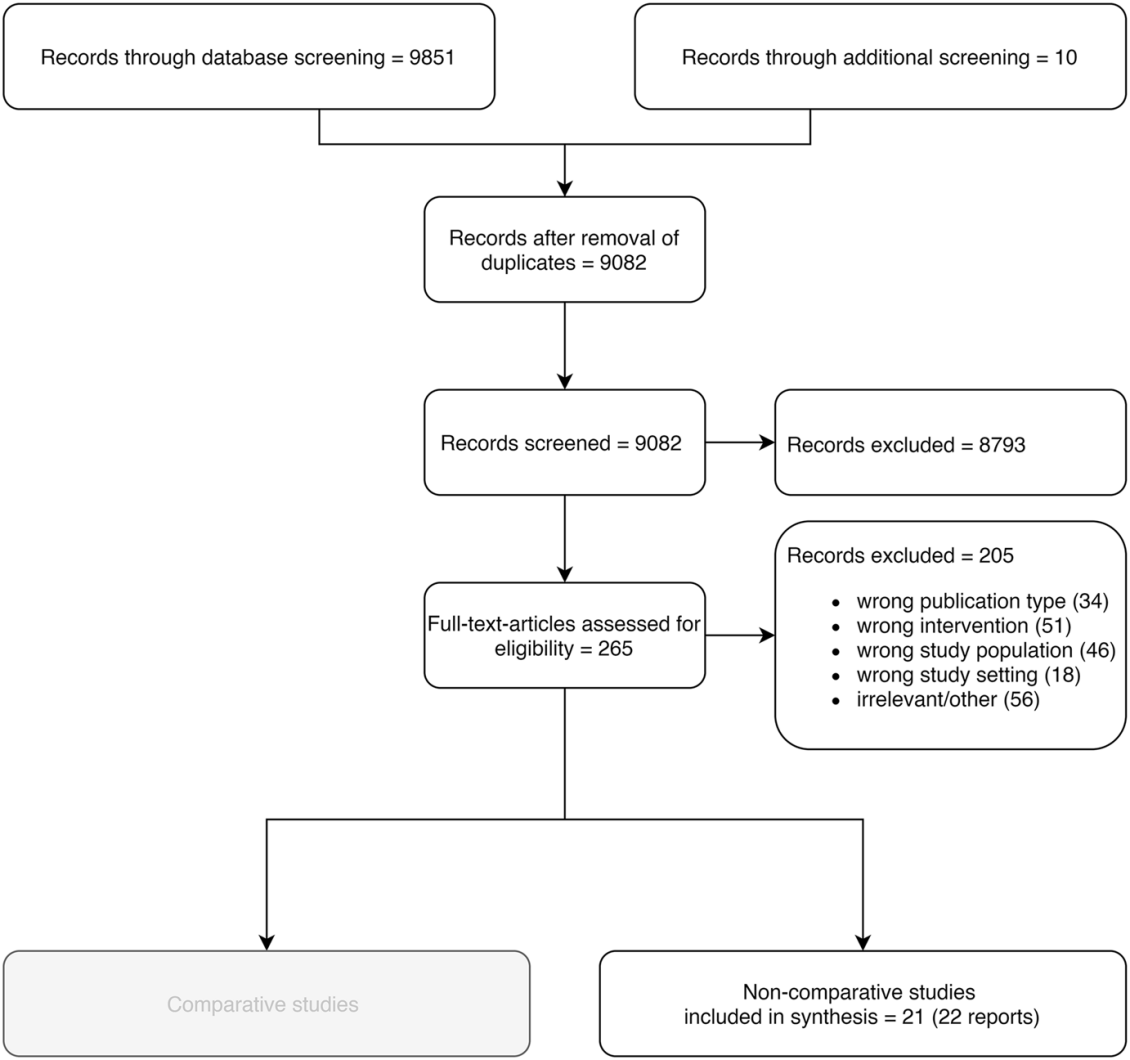
Flow of records through the screening process

Publication dates ranged from 2005 to 2020. The studies were conducted in Australia, Austria, France, Germany, Great Britain, Italy, Japan, Netherlands, New Zealand, Sweden, USA, and in one unspecified country. Devices used included care robots and personal robotic assistants, Ambient-assisted-living-Systems with integrated robotic components, Companion devices, and a smart-screen-assistant. The studies covered a total of 399 participants ranging from 3 to 51. With the exception of 2 studies (Cavallo et al., 2018; Torta et al., 2014), all participants had some kind of physical or cognitive impairment indicating a need for assistance. 5 studies investigated the use at home or in cohabitation settings (Baisch et al., 2018; Fattal et al., 2020; Frennert et al., 2017; Heerink, 2008; Khosla et al., 2021). 7 studies developed living lab settings (Bedaf et al., 2018; Cavallo et al., 2018; Heerink et al., 2009, 2010; Torta et al., 2014; Wade et al., 2011; Wu et al., 2014). 8 studies investigated different forms of group exercises (Bradwell et al., 2019; Lewis et al., 2016; Miyachi et al., 2017; Moyle et al., 2016; Robinson et al., 2016; Šabanović et al., 2013; Ujike et al., 2019; Kazuyoshi Wada et al., 2005; Wada et al., 2006) and one study investigated individual interaction (Pu et al., 2020). Methodologies included explorative qualitative research approaches, descriptive and correlative quantitative approaches, and different mixed-methods methodologies. Table 1 presents an overview. A critical appraisal of the methodological quality of each study according to MMAT can be found in Table 2.

**Table 1:** Study characteristics

Study	*n* =	Population	Add. groups	SAT	Setting	CN	Methods
Qualitative studies							
Bradwell et al.,( 2019)	17	Older adults in an aged care facility, aged 60–99	Roboticists (*n* = 18)	Paro, Joy for All Cat, Joy for All Dog Miro, Pleo, Perfect Petzzz Dog, Furby Hedgehog	Group exercise with different stations	GB	Content analysis of field notes of non-participant observation, focus groups
Frennert et al. (2017)	7	Older women living alone, aged 70—90 years	No	Hobbit	Cohabitation with a social robot	SE	Content analysis of semi-structured interviews and documents, observations
Pu et al. (2020)	11	Older adults in aged care facility with diagnosed dementia and mild to severe pain, aged 65–94	No	Paro	Individual free interaction	AUS	Inductive thematic analysis of semi-structured interviews
Ujike et al. (2019)	n/a	Older adults in aged care facility with schizophrenia	No	Pepper	Recreational group exercise	JP	Content analysis and video-analysis of interaction
Quantitative descriptive studies							
Cavallo et al. (2018)	45	Adults over 65	No	ERA robotic system	Living lab setting	IT	Cross sectional survey
Fattal et al. (2020)	15	Hospital patients undergoing rehabilitation aged 60–88	No	Pepper	Cohabitation with a social robot	FR	Longitudinal survey
Heerink (2008)	30	Older adults, aged 65–89	No	Steffi	Home use	NL	Cross sectional survey
Heerink et al. (2009)	30	Older adults living in or near elder care institution (65–94)	No	iCat	Living lab setting	NL	Cross sectional survey
Heerink et al. (2010)	40	Older adults living in an elder care institution, aged 65–89	No	iCat	Living lab setting	NL	Cross sectional survey
Miyachi et al. (2017)	51	Older adults in a care facility aged over 65	No	PALRO	Recreational group exercise	JP	Cross sectional survey
Šabanović et al. (2013)	10	Older adults in aged care facility with variying levels of physical and cognitive impairment	No	Paro	Group interaction in multi-sensory behavioural therapy session	US	Longitudinal observation
Wada et al. (2005)	10	Older women in aged care facility with varying degrees of cognitive impairment, aged 77–98	No	Paro	Group exercise	JP	Longitudinal survey
Wada et al. (2006)	10	Older women in aged care facility with varying degrees of cognitive impairment, aged 77–98	No	Paro	Group exercise	JP	Longitudinal survey
Wade et al. (2011)	7	Adults with physical impairment after stroke, aged 24 to 72 years	No	Bandit	Living lab setting	n/a	Cross sectional observation
Mixed methods							
Baisch et al. (2018)	43 in quantitative part, 3 in qualitative	Older adults over 65	Care providers (n = 30)	Paro, Pleo	Cohabitation with companion	DE	Cross sectional survey, semi-structured interview, non-participant observation
Bedaf et al. (2018)	10 in quantitative part, 9 in qualitative part	Older adults receiving home care, aged 62–93)	No	Care-o-bot	Living lab setting	NE	Cross sectional study supplemented with semi-structured interviews
Khosla et al. (2021)	5	Older adults with dementia	Family members	Betty	Home use	AUS	Cross sectional survey, video analysis, analysis of log data
Lewis et al. (2016)	6	Older adults in care facility with physical or cognitive impairment	Care providers (n = 5)	NAO-Bot, Paro	Group exercise with NAO-Bot as instructor	US	Cross sectional video analysis, focus groups
Moyle et al. (2016)	5	Older adults with varying forms of dementia, aged 68–78	No	CuDDler	Group exercise	AUS	Case study video analysis, thematic analysis of interviews
Robinson et al. (2016)	20	Residents of aged care facility with cognitive impairments	Staff members (n = 21)	Paro	Group exercise	NZL	Coding of observation and open-ended questions
Torta et al. (2014)	8	Older adults, cognitively healthy	No	KSERA Smart home with NAO-Bot	Living lab setting	AT	Cross sectional survey and qualitative interviews
Wu et al. (2014)	11 in quantitative, 7 in qualitative	Older adults, aged 76 – 85 years	No	Kompai Robot	Living lab setting	FR	Cross sectional survey, semi-structured interviews, focus groups

**Table 2:**
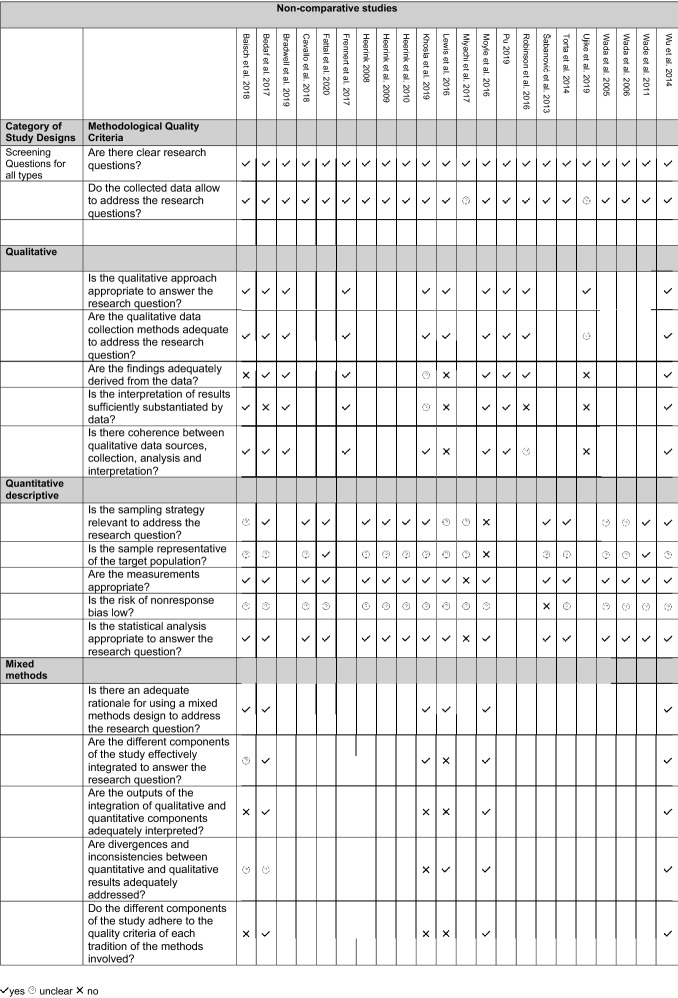
Critical Appraisal

In the following, we report on three major themes emerging from our synthesis. We call these themes (a) affective reactions and attitudes towards interaction, (b) results in regard to the perception of a social relation and quality of interaction, and, (c) results in regard to behavioral reactions and use.

### Affective Reactions and Attitudes Towards Interaction

This theme encompasses results concerned with emotional reactions of users during interactions with SATs as well as their general attitude towards interaction understood as a “tendency that is expressed by evaluating a particular entity with some degree of favor or disfavor” (Eagly & Chaiken, 2007). Several studies report overall positive attitudes of users (Bedaf et al., 2018; Cavallo et al., 2018; Fattal et al., 2020; Frennert et al., 2017; Miyachi et al., 2017; Moyle et al., 2016). This is indicated by a general openness and a certain curiosity to explore functions and to interact with SATs (Frennert et al., 2017; Wu et al., 2014). 3 Studies investigated acceptance, willingness, or intention to use and found a general tendency of approval towards sharing daily situations (Fattal et al., 2020; Heerink et al., 2009; Kazuyoshi Wada et al., 2005; Wada et al., 2006). Anxiety, hesitancy to interact, or otherwise negative emotional responses were found to be lower or were found to be voiced by a minority of participants (Cavallo et al., 2018; Fattal et al., 2020; Heerink et al., 2009). Positive emotional reactions such as relaxation, enjoyment, happiness, effects on the general mood and increased behavioral engagement on the other hand were frequently reported (Cavallo et al., 2018; Khosla et al., 2021; Lewis et al., 2016; Kazuyoshi Wada et al., 2005; Wada et al., 2006).

A smaller fraction of studies investigated influencing factors regarding emotional responses and attitudes. These studies suggest that attitudes of users are influenced by the perception of the functionality of the devices such as specific tasks that could be performed (Bedaf et al., 2018; Khosla et al., 2021; Miyachi et al., 2017). In addition, the perceived adaptability (Heerink et al., 2009, 2010) or flexibility (Šabanović et al., 2013) seems to play a role. Negative reactions were reported to occur especially in connection with malfunctions or if a gap between user expectations and functional capabilities was observed (Frennert et al., 2017; Moyle et al., 2016; Wade et al., 2011). 3 Studies indicate that the devices’ design decisively influences attitudes. Especially the perception of a social presence, for example, through humanoid appearance and users’ ability to interact seems to be important (Heerink et al., 2009, 2010; Pu et al., 2020). Perceiving a social dimension in the interaction is not only preferred by the users but is also shown to increase the accessibility as the perceived ease of use in interacting with SATs (Heerink et al., 2009; Torta et al., 2014; Ujike et al., 2019).

### Social Relations and Quality of Interaction

This connects to the second theme of findings which includes results regarding the quality and the effects of the interaction and how users experience the interaction with SATs. These findings suggest that the quality of interaction is not only determined by the functional capabilities but also by the perception of a social relation.

The dimension of social relations was investigated in a majority of studies in this subset (Bradwell et al., 2019; Cavallo et al., 2018; Fattal et al., 2020; Frennert et al., 2017; Lewis et al., 2016; Miyachi et al., 2017; Pu et al., 2020; Robinson et al., 2016; Ujike et al., 2019; Kazuyoshi Wada et al., 2005; Wu et al., 2014). This includes results concerned with experiences reported by users, for example through interviews and questionnaires as well as results observing and analyzing interactions from a third-person perspective focusing on behavioral cues that indicate the experience of a social relation.

Several studies report participants to commonly attribute internal states to SATs while interacting. This includes ascription of intelligence, emotional states such as kindness as well as internal wishes and desires or physiological needs (Frennert et al., 2017; Pu et al., 2020; Robinson et al., 2016; Kazuyoshi Wada et al., 2005). Depending on the characteristics of the device, 4 studies reported on the ascription of different social roles such as “assistant”, “friend”, “grandchild” or “pet” which led users to address the devices within these roles and to act accordingly (Lewis et al., 2016; Miyachi et al., 2017; Pu et al., 2020; Robinson et al., 2016; Kazuyoshi Wada et al., 2005). 3 Studies mentioned naming and the wish to own (Bradwell et al., 2019; Fattal et al., 2020; Kazuyoshi Wada et al., 2005) the device as an indicator of an emotional attachment that is based on the perception of uniqueness (Ujike et al., 2019). Changing of users’ behavior in order to care for the device, for example by kissing, hugging, or stroking it, or by attempts to reduce stress and strain from it and to give it “the place it deserves” were also reported (Fattal et al., 2020; Robinson et al., 2016). Interestingly, several studies that investigated the occurrence of social relations in more detail found that users engaging in such behavior or ascribing such properties were aware of the mechanical nature of the devices at the same time (Frennert et al., 2017; Pu et al., 2020; Robinson et al., 2016). Users were able to talk about the device as a technical instance or were able to evaluate its functioning as a machine. Only in one study, it was noted that participants were under the impression to interact with a “real animal” (Pu et al., 2020).

Understanding of the device and the context of the interaction was found to be an important influence on how users conceptualize and experience interacting with SATs. 4 Studies indicated problems of users to adequately understand technical details (Bedaf et al., 2018; Fattal et al., 2020; Frennert et al., 2017; Moyle et al., 2016). In regard to the social context, the perception of familiarity was noted as an important factor by some studies (Miyachi et al., 2017; Pu et al., 2020; Robinson et al., 2016; Wu et al., 2014). It seems to be the case that perceiving SATs as familiar allows users to situate the interaction within the boundaries of known territory and enables them to steer expectations accordingly.

### Behavioral Reactions and Use

4 Studies investigated the behavioral engagement of participants with the devices (Robinson et al., 2016; Šabanović et al., 2013; Torta et al., 2014; Wade et al., 2011). These studies show behavioral engagement to be stable over longer periods with a tendency to increase in frequency or duration. In 4 studies it was reported that interaction affected interactions with others such as care providers or family members (Fattal et al., 2020; Moyle et al., 2016; Robinson et al., 2016; Ujike et al., 2019). In these cases, the encounter with an SAT increased opportunity to get into contact with others. A theme constantly arising was that interactional and functional capabilities of the devices were found not always to match users’ preferences, needs and expectations, that is, what they wished or aimed to use the devices for. As 3 studies indicate, users may have different ideas about the relationship and how to make use of the devices, for example as to who should be in charge or in regard to the purpose and tasks a device should be equipped for (Bedaf et al., 2018; Bradwell et al., 2019; Cavallo et al., 2018; Fattal et al., 2020; Frennert et al., 2017). Consequently, results in regard to the benefits of use as perceived by users show mixed responses (Bedaf et al., 2018; Cavallo et al., 2018; Fattal et al., 2020; Frennert et al., 2017; Khosla et al., 2021; Moyle et al., 2016; Robinson et al., 2016; Torta et al., 2014). 4 studies investigating this dimension found that functions were often perceived as too limited by users to be of a clear use to the participants (Fattal et al., 2020; Khosla et al., 2021; Moyle et al., 2016; Torta et al., 2014). Functions that were already implemented otherwise in the participants’ surroundings were not used after initial exploring and, hence, provided no additional benefit (Frennert et al., 2017). In addition, several studies report technical difficulties to occur during their trials (Fattal et al., 2020; Frennert et al., 2017; Moyle et al., 2016; Torta et al., 2014). This was either due to the users’ initial understanding of how certain functions work or due to technical deficiencies and limitations of the devices themselves. 3 studies observed an interesting effect on users that is connected to the limitations of the devices. Users tend to be either frustrated as outlined above or alter their behavior and habits to suit the devices’ requirements (Fattal et al., 2020; Frennert et al., 2017; Wade et al., 2011). This could be, for example, to use a different voice or tone to avoid occurring problems with speech recognition, to confine oneself to the use of single functions, or change the way of approaching the device.

## Discussion

**Fig. 2:**
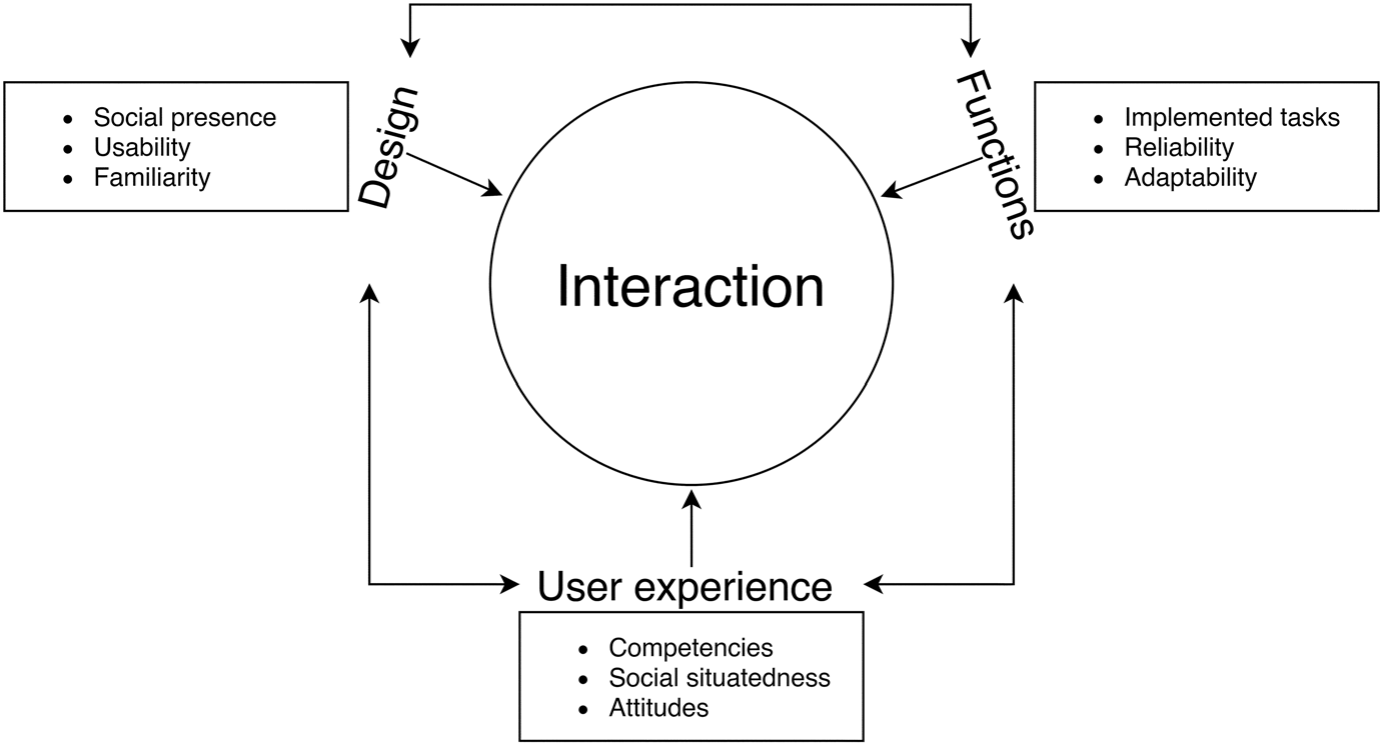
Schematized model of influences

The three main themes presented here, underline that interaction with SATs is shaped by the functional properties of the devices, their design, especially in regard to the appearance of the interface, and the context of use; that is, the users’ ability to understand the devices and to engage in interaction as well as their comprehension of the situation, for example in regard to the perception of social roles or own needs and preferences. Figure 2 shows the concept of interaction emerging from these themes.

The aim of this review was to provide an overview of existing studies and methodologies used, and to discuss their significance for the ethical debates. debates. From this normative perspective, the empirical knowledge gathered by the studies presented here is important as ethical deliberation on SATs falls within the domain of so- called mixed judgements (Düwell, 2009). Mixed judgments refer to normative principles as well as information about factual states of the world (empirical knowledge) to develop practically relevant and highly specific ethical conclusions (Ives et al., 2018; Mertz et al., 2014, Musschenga, 2005). The quality, adequateness, and practical relevance of such ethical arguments is largely dependent on the quality and content of the available factual knowledge. In this regard, our results add to three important dimensions of the ethical debate on SATs as they demonstrate the potential for certain pitfalls with commonly referenced empirical claims. This includes, first, methodological considerations in regard to the reliability and validity of empirical information that is used to inform ethical judgements. A second important point refers to the significance of empirical evidence in the debate on ethical acceptability of social interfaces. Finally, the evidence raises questions in regard to adequate concepts of autonomy based on the third theme. In the following, we will elaborate on these interpretations in detail. In what follows, we are going to comment on the significance of the empirical claims in these arguments in light of our findings.

### Methodological Quality

From a methodological perspective points to consider are the broad range of approaches to gathering and analyzing empirical data and implications in regard to the quality of the empirical evidence found. Empirically informed ethical considerations are reliant on detailed and reliable empirical information to develop judgements on the issue in question (Mertz et al., 2014). However, our review shows a broad range of different methods and methodological approaches used in this field. Approaches include qualitative, quantitative, and mixed-methods approaches sharing the common goal to develop and deepen the understanding of interaction with SATs. Methods employed for this purpose include in-depth interviews, surveying of participants, different kinds of observational methods, and analysis of additional sources such as logfile data. The impression of this variety is partly attributable to the methods of this review. However, a second and even more important reason might be that the studies included are situated in a variety of different academic fields following different perspectives, methodological traditions, and standards. Regarding quality, our appraisal shows diverse rigor of study designs and reporting ranging from low to high-quality reports. Despite the differences in academic traditions and research fields, we understand both quality and variety of the studies to limit the generalizability and applicability of the results in informing normative considerations. The research on interactional effects of SATs is still in its infancy. Careful consideration of empirical evidence is necessary to avoid inconclusive generalizations, misinterpretations, and biases.

A good example can be given in regard to our first theme (affective reactions and attitudes towards interaction). This theme gathers empirical evidence in regard to attitudes and affective reactions of users based on their experience with SATs. It shows an overall positive attitude, highlighting the participants face engagement with SATs open-minded, curious, and well aware of potential benefits. Especially in new research fields in which only little data exists, attitudes of stakeholders can be a valuable source for ethical arguments if understood as based on normative intuitions or basic preferences such as protection of self-interests or other basic values deemed worthy of protection (Bedke, 2008; Vandemeulebroucke et al., 2018b). They can, hence, be used to explore potential ethical caveats and to raise new arguments. They are also of viable interest to ethical design approaches aiming to foresee possible negative consequences (Vandemeulebroucke et al., 2018b).

The positive attitudes point to arguments in favor of using and furthering the development of SATs as it seems to be the case that stakeholders do not sense to be at risk of being violated in basic values. Such claims can be advanced further by pointing to the differences between studies including a general population (European Commission, 2012) and those which are based on actual experiences. While in the first case the attitude seems to be rather skeptical and hesitant, the actual experience seems to lead to a positive change (Baisch et al., 2018). Critical intuitions, hence, seem to be less stable, are suspected to build upon fictional ideas of technological care, and do not survive confrontation with reality (Frennert et al., 2017).

We do not deny general claims that experience actually changes attitudes. However, our critical appraisal indicates that such arguments need to be handled with caution. Nearly all studies we investigated are susceptible to a non-response bias in their selection of participants. A non-response bias is a distortion of data based on the sampling procedures or the procedures of data collection which can influence samples towards specific attitudes. As almost all studies in our subset used purposive methods of sampling or heavily relied on the participant’s willingness and curiosity to take part it should be no surprise that these samples show a more positive attitude and it is not unlikely that the noted overall positive attitude is attributable to such a distortion of the sample.

As a result, these considerations warrant a careful referencing of the existing empirical evidence. To our understanding, more research is needed to improve the methodological quality of respective studies. This should include rethinking recruitment methods to avoid biases. Considering different academic fields involved, more interdisciplinary approaches are worthwhile yet less often represented in this field. We also hold it is important to develop study designs that are able to capture the different perspectives included in different methodologies in a methodical way as all of them deliver important insights into different aspects of the interaction with SATs. A suitable way may, hence, be to further the development of mixed-methods approaches in which different perspectives and data sources are able to supplement each other and methodically contribute to a deepened understanding.

### Implications of Social Interfaces

Results on the quality of interaction especially connect to the question of ethical acceptability of social interfaces. On the one hand, our results support the assumption that social interfaces are valued by users to interact with respective devices and to get easy access to the supportive services these devices offer. This is in line with existing empirical evidence and ethical arguments highlighting the advantages of this way of interacting as a way to benefit from technological arrangements without extensive knowledge or technical abilities (Vandemeulebroucke et al., 2018b). However, ethical debates have also extensively discussed features of social interfaces against the background of manipulations and deceptions of users based on the misconception of SATs as a “real” social presence (Danaher, 2020; Sharkey & Sharkey, 2012; Sparrow & Sparrow, 2006). These arguments usually define deceptions as knowingly creating false beliefs and defend their moral inadequacy by pointing to the fact that a person’s beliefs and their congruence with the world are a necessary prerequisite to exercise one’s autonomy to a full extent (Matthias, 2015). Others have argued that knowingly creating false beliefs about the nature of technical devices such as SATs deprives persons of their right to be treated as an end in themselves and is, hence, a violation of dignity (Decker, 2008; Sparrow & Sparrow, 2006). These arguments deserve special attention in the use of SATs in healthcare as it is usually directed towards specifically vulnerable groups, that is, persons whose condition (e.g. health, age) indicates an elevated risk of being violated in their moral goods or interests (Boldt, 2019).

Our review confirms assumptions regarding the vulnerability of user groups. This is shown by the respective study populations. Concerning the question of morally problematic misconceptions that could be classified as deceptions, the empirical data seems to indicate frequent occurrences at a first glance. This applies with respect to the ascription of internal states, wishes, and desires by users as well as regarding behavioral cues indicating that users might find themselves in a relationship with a real social being. The problem of deception, hence, seems to be far away from being a merely theoretical or philosophical problem. However, the data also indicates that matters might be more complicated. For example, several studies explicitly commented on their results or highlighted within their findings that users, while showing respective behavior or ascribing respective states to the devices, were well aware of the technical nature. This was indicated by, for example, being able to address the technical level of the device, evaluating its performance as a machine, or exercising respective behavior towards the device.

Similar findings, for example by Nass and Moon (2000), have described this behavior as part of media- or technological competencies, that is, the ability to interact with technological artifacts “as if” they were social entities while at the same time being perfectly aware that the counterpart is a technical arrangement and not an animate being. If this is the case, our findings neither confirm nor disprove arguments on manipulation and deception but warrant refinement of these arguments in line with this empirical evidence. First, the evidence implies that not every observable behavior or respective ascription is a sign of a misconception in the sense of deception. Second, and even more important, being able to interact with devices on these two levels (“as if” and as device) is a necessary competence to profit from a social interface while avoiding falling for false beliefs as outlined above. This includes the ability to transcend the technical nature of the devices, to understand its social presence as part of its interface, and to be able to decide to communicate on this level.

Regarding the former, this indicates the need for more research to understand how users experience interactions with SATs and how they manage two different levels of these interactions and not fall for deception. A more detailed description of these competencies would be required. Secondly, the empirical results indicate that ethical arguments regarding deceptions and manipulations have to be revisited to include the crucial role of the aforementioned competencies as a probable dividing line between the useful and easy access to complex technical structures and ethically inadmissible deception. This is especially important as technical literacy that would be presupposed as part of these competencies surely varies with the cognitive abilities of users and, hence, would also be part of the possible decline of these abilities in case of certain diseases.

### Questions of Relations and Autonomy

Finally, our results indicate that functionality of devices is often limited or is perceived to be limited due to a lack of understanding. In these cases, users either become frustrated or tend to align their way of acting and interacting with the real or perceived limitations of their SAT.

From a medical ethics perspective focusing on autonomy, this finding raises important questions. It has been argued on various occasions that SATs are devices to enhance autonomy of their users. They allow, for example, to maintain ways of acting and satisfy important needs that would not have been possible to exercise otherwise or would have been left unsatisfied without them. Our findings, however, indicate that this improvement in user autonomy comes at certain costs. Especially due to limited functionality or limited understanding, users have to adapt to the technical rationality of the devices to be able to make use of them. Despite all efforts in user-centric designs in recent years, it seems to be the case that users often change behavior, habits, and patterns to suit their SAT, not vice versa. From a perspective of autonomy, this has to be understood as a source of concerning losses in determining own choices which need to be carefully balanced against possible benefits connected to the use.

In ethical debate, this aspect has been largely overlooked so far. This is the case, first, as most empirical study designs follow a classic “interventionist paradigm”, neglecting the impact of people’s behavior and handling on technology itself. This approach is critically assessed by new theories on co-constitution of age and technology (Peine & Neven, 2019). Secondly, prominent concepts of autonomy in healthcare often rely on an individualistic perspective. These concepts focus on abilities and competencies of an individual to act and decide freely and are, hence, concerned with the capacities of an independent decision-maker and the absence of external influences on these capacities. Yet, broader accounts of autonomy have stressed that social surroundings and relations of decisions and decision-makers play an important role in determining an agent’s ability to decide. These accounts draw attention to social conditions which may limit or foster a person’s ability to decide and pursue their goals and preferences. Aligning to the technical rationality of a device to make use of it provides an illustrative example of such influences. In this regard, our findings suggest reconsidering the concepts of autonomy used in this debate to be able to adequately consider such relational and conditional effects.

## Conclusion

Based on a comprehensive review, we have synthesized existing empirical evidence on relational and interactional effects of SATs in healthcare with the aim of evaluating the significance and impact of existing empirical knowledge on ethical arguments concerned with SATs.

In regard to such empirically informed ethical judgments, our review shows certain pitfalls and potential problems of commonly made arguments. First, it seems to be important to be cautious about the reliability and validity of empirical claims that can be drawn from studies on SATs. Our quality appraisal shows a very diverse landscape. An important limitation of the data is a ubiquitous selection bias found in most of the studies. Secondly, we have highlighted the need to consider the role of empirical evidence in arguments on manipulation and deception through social interfaces. Evidence indicates that these arguments warrant further refinement as their assumptions do not seem to capture the reality of human-machine-interaction. Finally, our results indicate a new facet of arguments on autonomy and use that has been largely overlooked so far.

### Strength and Limitations

With this, we present – to our knowledge – the first comprehensive review of empirical evidence on interactional and relational effects of SATs and its impact on the ethical debate. We have to concede, however, that this work comes with certain limitations. First, one has to be aware that this report covers only evidence in non-comparative study designs while data on comparative study designs is reported separately. This way of reporting is based on the assumption that empirical statements drawn from different types of studies play a different role in ethical arguments and, hence, should be treated separately. Nevertheless, this implies that this report does not cover all available evidence nor is it able to assess the impact on all ethical arguments and cannot provide a complete overview. Secondly, one has to be clear that our considerations and conclusions should not be treated as generalizable statements or as arguments for or against a specific technology or SATs in general. As the included studies vary in used artefacts, settings, population’s cultural backgrounds, and other aspects, it is neither adequate nor possible to generate such statements. It has to be noted, however, that the goal of our work was not to infer such generalizations but to give an overview of the existing empirical data and assess its impact on existing ethical arguments. With this in mind, our work shows the potential for certain pitfalls and unwarranted claims, one needs to be aware of. Whether these problems apply to arguments made toward a specific technology would then be a matter of further and detailed inspection but is not part of our work here.

## Data Availability

The datasets generated and analysed during the current study are available from the corresponding author on reasonable request.
